# Histaminergic Modulation of Cholinergic Release from the Nucleus Basalis Magnocellularis into Insular Cortex during Taste Aversive Memory Formation

**DOI:** 10.1371/journal.pone.0091120

**Published:** 2014-03-13

**Authors:** Liliana Purón-Sierra, María Isabel Miranda

**Affiliations:** Departamento de Neurobiología Conductual y Cognitiva, Instituto de Neurobiología, Universidad Nacional Autónoma de México, Querétaro, Qro., México; Sapienza University of Rome, Italy

## Abstract

The ability of acetylcholine (ACh) to alter specific functional properties of the cortex endows the cholinergic system with an important modulatory role in memory formation. For example, an increase in ACh release occurs during novel stimulus processing, indicating that ACh activity is critical during early stages of memory processing. During novel taste presentation, there is an increase in ACh release in the insular cortex (IC), a major structure for taste memory recognition. There is extensive evidence implicating the cholinergic efferents of the nucleus basalis magnocellularis (NBM) in cortical activity changes during learning processes, and new evidence suggests that the histaminergic system may interact with the cholinergic system in important ways. However, there is little information as to whether changes in cholinergic activity in the IC are modulated during taste memory formation. Therefore, in the present study, we evaluated the influence of two histamine receptor subtypes, H_1_ in the NBM and H_3_ in the IC, on ACh release in the IC during conditioned taste aversion (CTA). Injection of the H_3_ receptor agonist R-α-methylhistamine (RAMH) into the IC or of the H_1_ receptor antagonist pyrilamine into the NBM during CTA training impaired subsequent CTA memory, and simultaneously resulted in a reduction of ACh release in the IC. This study demonstrated that basal and cortical cholinergic pathways are finely tuned by histaminergic activity during CTA, since dual actions of histamine receptor subtypes on ACh modulation release each have a significant impact during taste memory formation.

## Introduction

The central cholinergic system has long been associated with many aspects of cognitive functioning and there is clear evidence implicating cholinergic neurons in the mediation of learning and memory processes [1]–[3]. Acetylcholine (ACh) is released in the neocortex in response to a variety of behavioral and environmental conditions [4]–[10].

The processing of a gustatory stimulus is very complex in and of itself because taste encoding encompasses both the immediate hedonic value of a tastant and one's overall experience with the perceived flavor. Accordingly, a gustatory memory is a representation not only of the explicit characteristics of a taste, but also of its degree of familiarity. There is extensive evidence pointing to the importance of cholinergic activation as a marker of novelty. The role of ACh in the recognition of novel taste is well documented [11]–[15] and consistent with ACh's established involvement in the encoding of other types of novel-experience memories [4], [16]–[19].

Conditioned taste aversion (CTA) is a gustatory conditioning paradigm in which subjects learn to avoid a particular taste when it is followed by gastric malaise [20]. During CTA acquisition, presentation of a novel taste, but not a familiar taste, increases ACh release in the insular cortex (IC), a central structure in taste recognition memory. Cholinergic cortical activity during taste memory acquisition differs from that during taste memory retrieval, supporting the notion that ACh may facilitate cortical plasticity during memory formation [5], [13]. Moreover, there is a requirement for modulation of cholinergic cortical release and an intact nucleus basalis magnocellularis (NBM) in the cholinergic basal forebrain during the early stages of learning, but no such requirement during memory recall [4]–[7]. Notably, we demonstrated previously in the CTA paradigm that novel taste consumption elicits a significant increase in ACh release in the IC and that tetrodotoxin infusions into the NBM, which disrupt NBM-mediated release of ACh into the cortex and basolateral amygdala, impair acquisition, but not retrieval, of CTA [5].

This inverse correlation observed between familiarity and cholinergic activity [5], [13] is congruent with the view that cortical ACh enhances responsivity to afferent sensory input while decreasing internal processing related to previously formed cortical representations [21]. At the behavioral level, the actions of ACh could be interpreted as an enhancement of attention or memory encoding [10], [22]–[24]. In other words, cortical ACh could be supporting new stimuli encoding processes, at least in part, by reducing interference from previous memories, as has been suggested to occur in hippocampal circuits involved in long-term memory formation [23], [25]–[28]. Hence, NBM-cortical cholinergic interactions could be playing a similar role in taste recognition memory.

Although ACh has been shown to modulate many other neurotransmitter systems, new studies suggest that the cholinergic system itself may receive modulation from the histaminergic system [6], [29]–[32]. The NBM receives histaminergic afferents directly from the tuberomamillary nucleus in the posterior hypothalamus, the main source of histaminergic projections and histamine is distributed throughout the entire cortex by histaminergic forebrain projections [33], [34]. Histamine's broad presence in the forebrain is consistent with suggestions that it may act as a neuromodulator during memory formation [35]–[44] Furthermore, in a recent study, we demonstrated opposite roles for subtype 1 (H_1_) and subtype 3 (H_3_) histamine receptors in the NBM and the IC, respectively, two areas important for taste memory. That is, blockade of H_1_ receptors in the NBM or activation of H_3_ receptors in the IC impaired CTA. These results demonstrated complementary roles for H_1_ and H_3_ receptors in CTA that could be the result of cortical cholinergic activity modulation [45].

In the present study, we evaluated whether activation of the NBM via H_1_ receptors or inhibition of the IC via H_3_ receptors affects cortical ACh release and influences taste memory formation. The presence and co-localization of H_1_ and H_3_ receptors in the NBM and IC, respectively, were determined. The effects of intra-IC injections of the H_3_ receptor agonist R-α-methylhistamine (RAMH) and of intra-NBM injections of the H_1_ receptor antagonist pyrilamine, during acquisition, on long-term CTA memory were evaluated. Changes in ACh release in the IC were assessed by microdialysis in free-moving animals during both the acquisition and retrieval trials.

## Materials and Methods

### Animals

Fifty-five male Sprague-Dawley rats, weighing 250–300 g at the time of surgery before CTA or sacrifice for immunofluorescence assays, were obtained from the Instituto de Neurobiología breeding colony. Rats were placed in individual acrylic cages and maintained at 23°C under an inverted 12-h/12-h dark/light cycle; all behavioral protocols were implemented during the dark phase. Food and water were available *ad libitum* until the behavioral procedures began. The experiments were performed in accordance with the Mexican Laws for Animal Care (Norma Oficial Mexicana SAGARPA) and the relevant rules set forth by the Mexican Ministry of Health. The experimental protocol was approved by our local Animal Care Committee (Comité de Bioética del Instituto de Neurobiología de la UNAM) and confirmed to be in compliance with the National Institutes of Health Guide for the Care and Use of Laboratory Animals (NIH publication 80-23, revised 1996).

### Immunofluorescence assays

For the immunofluorescence analysis, 6 rats were overdosed with pentobarbital (60 mg/kg) and perfused transcardially with saline (0.9%) and fixative solution (4% paraformaldehyde in 0.1 M phosphate buffer [PBS]). The brains were cryoprotected in 30% sucrose solution, and 40 µm coronal sections of each whole brain were obtained in a cryostat (Leica CM1850, Microsystems Inc., Buffalo Grove, IL). Sections containing the IC or NBM of the same brain were placed on separate slides (Superfrost/Plus by Thermo Fisher Scientific Inc., USA) and stored at 4°C until processing. For double immunofluorescence, the slices were washed in PBS-Tween 20 and blocked with a solution containing 2% donkey serum.

After blocking, the IC slices were incubated at 4°C for 24 h with the following primary antibodies: monoclonal mouse anti-GAD65 [1∶100] (Santa Cruz Biotechnology, Dallas, TX) and goat anti-H3R [1∶200] (Santa Cruz Biotechnology). The sections were washed in PBS and incubated for 2 h at room temperature with Alexa Fluor 488 rabbit anti-mouse IgG (Invitrogen, Life Technologies, USA) and Texas Red bovine anti-goat IgG (Santa Cruz Biotechnology, Dallas, TX) secondary antibodies, both at [1∶100]. The slices were washed in PBS again and incubated with 4′,6-diamidino-2-phenylindole (DAPI) nuclear counterstain (Vector Laboratories, Burlingame, CA) for 25 min.

Meanwhile, the blocked NBM slices were incubated with the following primary antibodies for 24 h at 4°C: monoclonal mouse anti-choline acetyl transferase (ChAT) [1∶100] (Santa Cruz Biotechnology, Inc., Dallas, TX) and rabbit anti-H1 receptor [1∶200] (Sigma, H6913, by Sigma Co., USA). Secondary antibody incubation was as described above except that Cy2-conjugated goat anti-rabbit [1∶100] and Cy3-conjugated donkey anti-mouse [1∶100] antibodies (Jackson immunoresearch Laboratories, Inc., West Grove, PA) were applied. All slices were washed and mounted with Vectashield H-1000 (Vector Laboratories, Burlingame, CA).

For imaging, a Zeiss LSM 780 Meta confocal microscope (Carl Zeiss, Germany) with a 40× oil-immersion objective was used. We applied 488 nm stimulation to excite Alexa 488 and Cy2, 561 nm stimulation to excite Cy3 and Texas Red, and 750 nm stimulation to visualize DAPI. For quantitative image analysis, z-stack images (4 or 5 consecutive 512×512 confocal sections) were obtained every 5 µm (stack size of 450 µm in the X and Y dimensions) and processed in Aim Image Examiner software. ImageJ analysis software (http://rsbweb.nih.gov/ij/) was used for quantitation of double immunolabeling. The population of cells labeled by each fluorescent probe was measured independently and contrasted with the number of DAPI labeled nuclei in the IC or the number of ChAT-positive cells in the NBM.

### Cannulation

**Figure 1:**
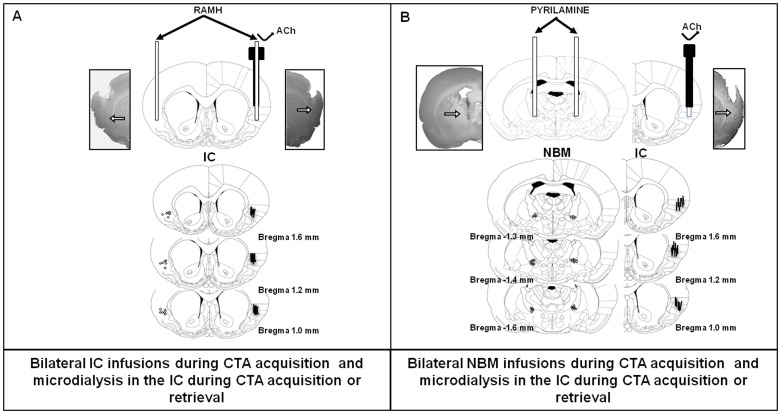
Coronal section diagrams and representative photomicrographs of the IC and NBM. Arrows in photomicrographs and dots in atlas diagrams show locations of stainless-steel cannulae; lines shows microdialysis probe trails. **A**) One stainless-steel cannula implanted in the left IC and one dual probe (with injector and cannula) implanted in the right IC. **B**) Stainless-steel cannulae placed bilaterally in the NBM and one cannula/microdialysis probe in the right IC.

Animals were anaesthetized with intra-parenchymal (i.p.) injections of ketamine (70 mg/kg) and xylazine (6 mg/kg) and submitted to standard stereotaxic procedures. One set of rats was implanted with a single 23 gauge stainless steel cannula aimed at 2 mm above the left insular cortex (1.2 mm anterior, 5.5 mm lateral and 3 mm ventral to Bregma; injector protruded 3 mm from the cannula) and a microdialysis guide cannula with an infusion tube (BASi MD 2262) in the right IC (corresponding coordinates for opposite side, and 4.9 mm ventral to Bregma; probe protruded 2 mm from the cannula) [46] (Fig. 1A). Another set of rats was implanted with two (bilateral) stainless steel cannulae aimed at 2.5 mm above the NBM (1.5 mm posterior, 2.5 mm lateral and 4.9 mm ventral to Bregma; injectors protruded 2.5 mm from the cannulae) and one microdialysis guide cannula (BASi MD 2200) in the right IC (coordinates above) (Paxinos and Watson, 2004) (Fig. 1B). The infusion cannulae and microdialysis guide cannula were fixed to the skull with two surgical screws and dental acrylic cement. Stylets were inserted into the guide cannulae to prevent clogging.

### CTA

Five days after surgery, after they had recovered completely, the rats were water deprived for 18 h, and then handled (∼3 min/d) and habituated over 4 d to drinking water for 15 min from a graduated bottle to get stable baseline water consumption data. The next day, CTA acquisition training was applied as reported elsewhere [5], [13], [47], [48]. Briefly, a novel sweet taste (0.1% saccharin solution) was presented for 15 min and, 45 min after the end of the drinking period, each rat was injected with LiCl (0.3 M, 10 ml/kg. i.p.) for induction of gastric malaise. CTA memory was tested 24 h later by re-presenting 0.1% saccharin solution. All CTA procedures (i.e. water baseline, acquisition, and retrieval) were done in the microdialysis chamber. Water and saccharin consumption volumes were recorded. An aversive taste memory was considered to be present if animals significantly decreased their consumption relative to acquisition.

### Microdialysis

**Figure 2:**
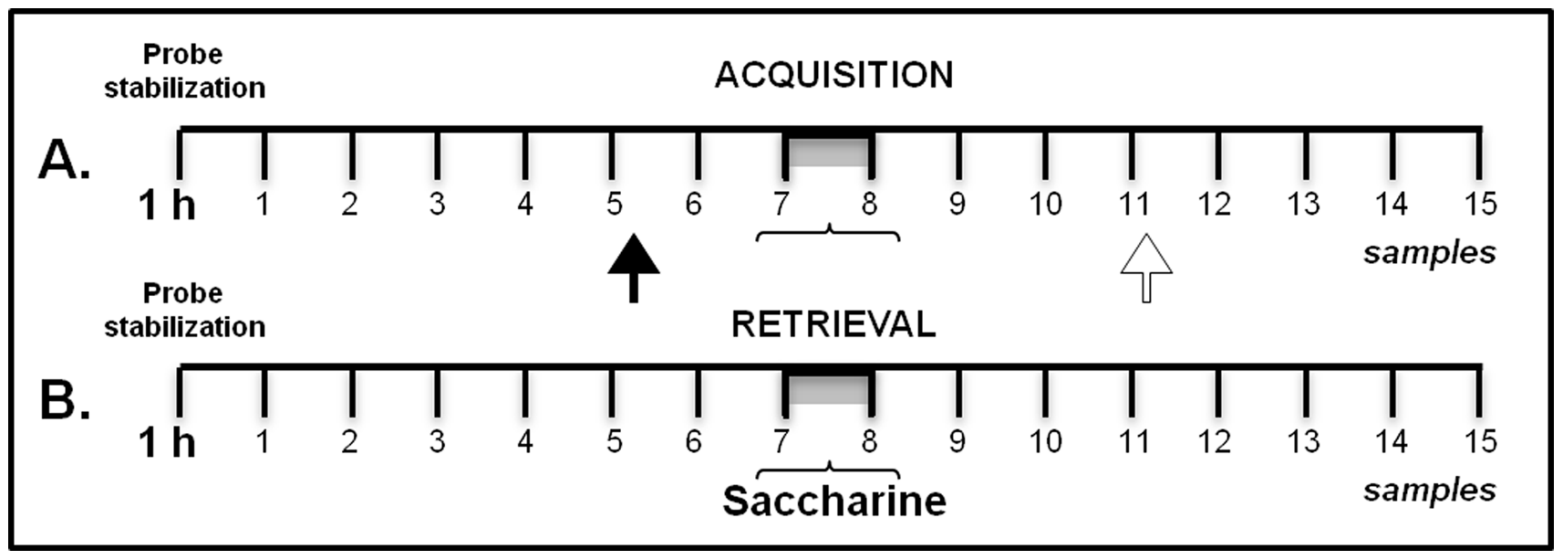
Summary of microdialysis ACh sampling protocol in CTA behavioral paradigm. Microdialysis samples were collected every 15-moving rats. **A**) During CTA acquisition, RAHM, pyrilamine, or saline was injected at the beginning of sample 5 (dark arrow), 30 min before saccharin consumption (*gray bar*), and LiCl was injected i.p. at the beginning of sample 11 (white arrow), 45 min after the saccharin consumption period had ended. **B**) During the CTA memory trial, ACh levels were measured in the absence of any injections.

As summarized in Figure 2, microdialysis performed during CTA acquisition or during CTA retrieval was conducted in eight independent groups. First, bilateral injectors were inserted into the stainless steel guide cannulae aimed at the NBM or a single injector was inserted into the unilateral guide cannula directed to the right IC (Fig. 1A–B). Second, dialysis was started by connecting the probe inlet (BASi MD 2200 or MD 2262 dialysis probes with 2 mm membrane) to a microinfusion pump system (CMA Microdialysis, West Lafayette, IN) that circulated the probe continuously with Ringer's solution (118 mM NaCl 4.7 mM KCl 2.5 mM CaCl_2_, and 10 µM neostigmine) at a rate of 2 µl/min to avoid ACh degradation, as described previously [5], [49]. The circulating microdialysis probe was inserted into the guide cannula directed at the right IC, and then the rat was placed in the microdialysis chamber. The initial 60-min sampling solution was discarded, and then samples were collected every 15 min.

Fifteen microanalysis samples were collected from right IC during CTA acquisition (see Fig. 2 for procedure summary). Each consecutive sampling period was 15 min. The NBM or IC microinfusions were started right before collecting sample 5. Thirty minutes (2 samples) later, 0.1% saccharin was presented along with sample 7. An i.p. LiCl injection was given after a 45-min saccharin consumption period, right before collection of sample 11. An additional four samples were collected before the microdialysis procedure was concluded. The same sampling procedure was repeated during CTA retrieval, except that no infusions or i.p. injections were administered (Fig. 2B). Immediately upon being collected, all microdialysis samples were frozen at −70°C or submitted to high-performance liquid chromatography (HPLC) analysis.

### Drug infusions

Independent microdialysis groups were used during the CTA acquisition versus retrieval days (Fig. 1, bottom). In preparation for the microinfusions, patency stylets were removed and 30-gauge injection needles were inserted into the cannulae. The injection needles protruded 2.5 mm and 2.0 mm beyond the cannulae for the NBM and IC injections, respectively. The injectors were connected via polyethylene tubing to two 10 µl microsyringes driven by an infusion pump (Carnegie Medicine, Stockholm, Sweden). During CTA acquisition, the SALINE (vehicle control) groups were given 0.5 µl microinfusions of saline (NaCl 0.9%) into the NBM or IC, the PYRILAMINE groups were given bilateral 0.5 µl microinfusions of pyrilamine (100 mM, Sigma-Aldrich) into the NBM, and the RAMH groups were given unilateral microinfusions of RAMH (10 µM, Sigma-Aldrich) into the right IC via the stainless steel cannula and into the left cortex via a microdialysis probe attached to the infusion tube (see Fig. 1A). The total volume of solution (0.5 µl per side of pyrilamine, RAMH, or saline) was delivered over 1 min. Dose of pyrilamine and RAMH were based on previous behavioral studies which demonstrated the modulating role of histaminergic receptors on different learning tasks [42], [50], [53], [54]. Injection needles were left inside the cannulae for one additional minute to allow diffusion of the injected solution into the tissue and to minimize dragging of the liquid back along the injection track.

### Determination of ACh levels

The microdialysis samples were injected into a polymeric reversed-phase column (BASi). ACh was assayed in the dialysate by HPLC with electrochemical detection using an ACh/choline chromatographic assay kit (BASi, West Lafayette, IN) consisting of an ACh analytical column (BASi MF-6150) and an ACh/choline immobilized enzyme reactor (IMER, BASi MF-6151). The mobile phase had a 1 ml/min flow rate and consisted of a 50 mM sodium phosphate buffer (pH 8.5) supplemented with 0.05% Kathon reagent (BASi, West Lafayette, IN), a broad spectrum antimicrobial suitable for enzyme preservation. ACh, separated in the analytical column, was hydrolyzed by acetylcholinesterase in the IMER into acetate and choline, which was then oxidized by choline oxidase into betaine and hydrogen peroxide. Hydrogen peroxide was detected electrochemically by a platinum-working electrode at +500 mV with an Ag/AgCl reference electrode. The sensitivity limit was approximately 0.1 pmol, and the signal/noise ratio was greater than 2. To evaluate the amount of ACh in each sample, a linear regression curve was made with ACh standards, and the peak areas of the compound in the samples were compared with those of the standards. ACh levels in the dialysate samples were calculated as pmol/15 min, and were not corrected for probe recovery (∼60%).

### Histology

One day after completing the microdialysis procedures, the animals were anaesthetized deeply with pentobarbital and perfused transcardially with 4% formaldehyde in 0.9% saline. The brains were placed in fresh formaldehyde overnight and then transferred to a 30% buffered sucrose solution and stored at 4°C. Coronal sections (50 µm thick) taken through the areas where the microdialysis probe and injectors had been were stained with cresyl violet and inspected under stereoscopic light (Fig. 1). Only data from animals with injector/probe tips located within the NBM and IC were included in the analysis.

### Statistical analysis

The inter-group differences in consumption during CTA acquisition and retrieval were determined by repeated measures analyses of variance (ANOVAs) followed by Fisher's *post hoc* tests. *P* values <0.05 were considered significant, and all datum values are expressed as means ± standard errors of the mean (SEMs). To compare ACh levels, repeated measures ANOVA was carried out with the extracellular ACh level data (pmol/20 µl) from samples 1–15. To analyze the source of detected differences, a simple, one-way ANOVA between groups for each sample or paired t-test for each group samples, was performed when appropriate.

## Results

### Verification of probe placement

Only animals confirmed to have their guide cannulae and microdialysis probe in the NBM (bilaterally) and IC were included in the data analyses (Fig. 1). Thirteen cannulated animals were excluded due to misplacement of cannulae/injectors or the probe.

### H_3_ receptors co-localize with IC GABAergic cells and H_1_ receptors co-localize with NBM cholinergic cells

**Figure 3:**
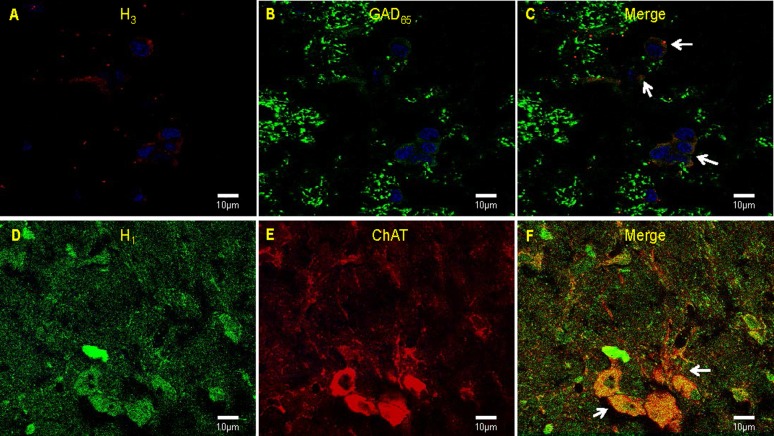
Confocal fluorescence microscopy images in the IC and NBM. Representative pictures of (**A**) H_3_ immunopositivity, (**B**) GAD_65_ immunopositivity, and (**C**) a merged image showing the co-localized expression of H_3_ receptors and GAD_65_ in the IC. Arrows point to H_3_/GAD double-labeled cells that where quantified with respect to DAPI-labeled nuclei (blue). Representative pictures of (**D**) H_1_ immunopositivity, (**E**) ChAT immunopositivity and (**F**) a merged image showing the co-localized expression of H_1_ receptors and ChAT. Arrows point to H_1_/ChAT double-labeled cells.

Immunofluorescence conducted in six brains revealed that 85% of IC cells were positive for H_3_ receptors and that 50% of those cells were also reactive to anti-GAD_65_, indicating that H_3_ receptors are expressed in GABAergic cells in the IC (Fig. 3A–C). The H_1_ receptor was detected in 70% of ChAT-positive NBM cells, indicating that H_1_ receptors are expressed ubiquitously throughout the NBM cholinergic cell population (Fig. 3, D–F).

### CTA-impairing RAMH injections into the IC alter ACh release during CTA acquisition and retrieval

**Figure 4:**
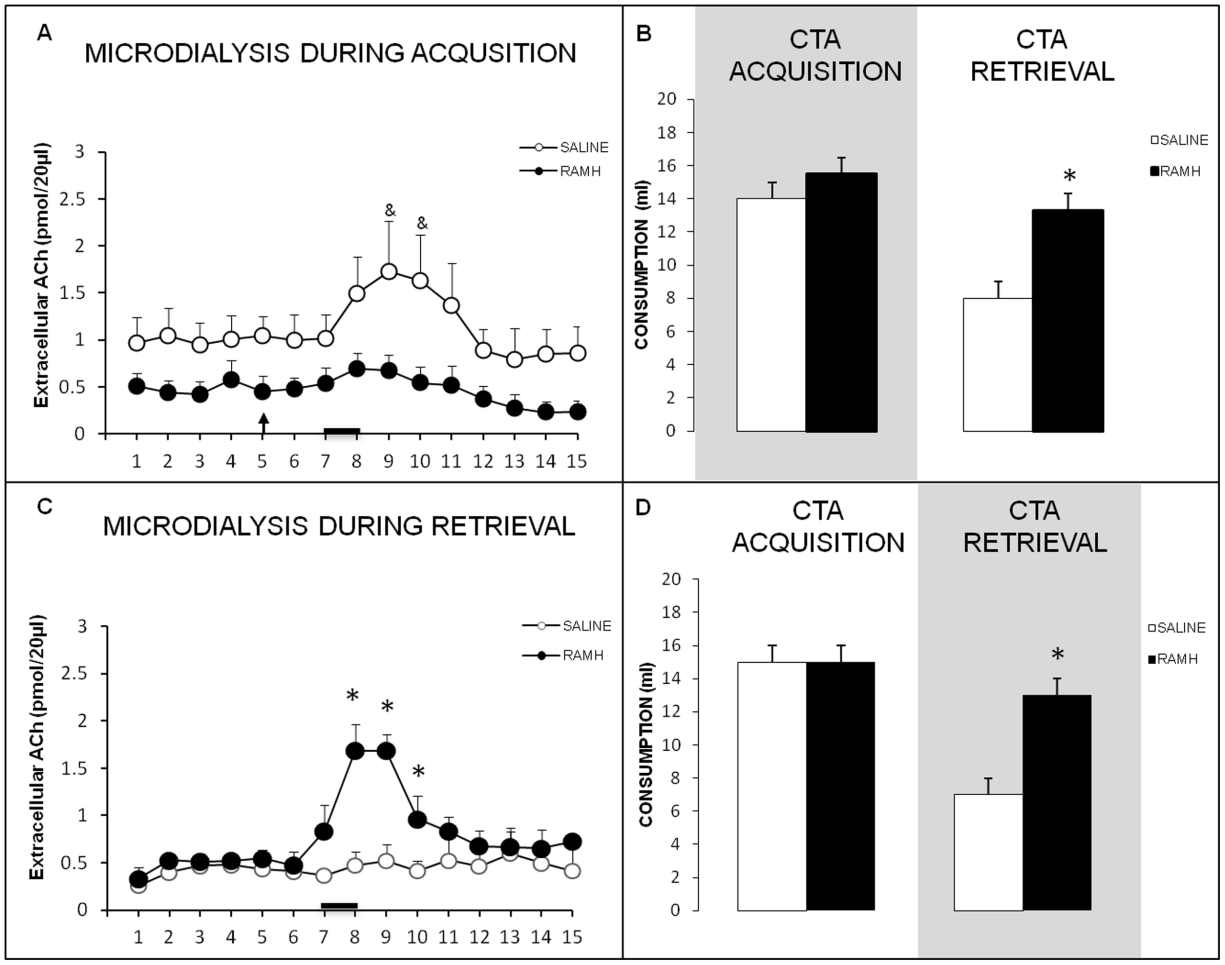
RAHM effects in ACh release during CTA acquisition and retrieval. **A**) Extracellular ACh in the IC of free-moving rats during CTA acquisition. The arrow shows time of saline or RAMH infusion, and the black bar indicates the saccharin consumption period (^&^ *p*<.05 vs. samples samples 2 and 3). **B**) Saccharin consumption during CTA acquisition (shading = microdialysis day) and memory retrieval (N = 4 for each group; *mean ± SEM*, *p<.05). **C**) Extracellular ACh in the IC of free-moving rats during CTA retrieval. The black bar indicates the saccharin consumption period (*p<.05). **D**) Saccharin consumption during CTA acquisition and memory retrieval (shading = microdialysis day). (N = 5 for each group; *mean ± SEM*, *p<.05).

As shown in Figure 4A, a repeated measures ANOVA indicated that ACh levels during CTA acquisition differed between samples (F_1,6_ = 3.465, *p*<.01), but did not differ between groups (F_1,6_ = 2.703, *p*>.05) and there was not a significant group×sample interaction (F_1,6_ = 0.425, *p*>.05). Paired t-test for each sample revealed that the sample effect was due to significant differences between the amount of ACh released in control group, in samples 9 and 10 relative to the basal ACh levels in samples 2 and 3 (*p*<.05).

H_3_ receptor activation by RAMH during CTA acquisition affected saccharin consumption in the retrieval trial only (Fig. 4B). A one-way ANOVA showed no significant differences in baseline water consumption between the groups (data not shown). A repeat measures ANOVA for saccharin solution consumption during acquisition and retrieval days revealed significant effects of group (F_1,6_ = 23.059, *p*<.01) and of experiment day (F_1,6_ = 19.443, *p*<.05), but not a significant interaction between these two factors (F_1,6_ = 3.722, *p*>.05). Fisher's *post hoc* tests indicated that saccharin consumption on the CTA acquisition day did not differ significantly among the groups (*p*>.05), indicating that motivation to drink and liquid consumption were unaffected by the intra-IC RAMH microinfusions. Conversely, *post hoc* analysis showed that consumption during CTA retrieval did differ significantly between the groups (*p*<.01). Animals that received intra-IC RAMH injections during CTA acquisition consumed significantly more saccharin solution during retrieval than did saline controls, indicating that the RAMH treatment impaired CTA memory formation.

The levels of ACh detected in the IC during memory retrieval in the control and RAMH groups (injections during CTA acquisition and microdialysis only during retrieval) are reported in Figure 4C. A repeated measures ANOVA for ACh levels during the retrieval trial revealed significant group (F_1,8_ = 8.01, *p*<.05) and sample (F_1,8_ = 43.350, *p*<.01) effects, as well as a significant group×sample interaction (F_1,8_ = 12.150, *p*<.01). A One-way ANOVA for each sample revealed higher ACh levels in the RAMH group than in the saline control group in sample 8 (F_1,8_ = 15.775, *p*<.01), sample 9 (F_1,8_ = 12.456, *p*<.01), and sample 10 (F_1,8_ = 7.031, *p*<.05); these three samples were subsequent to presentation of the taste stimulus at the beginning of the seventh sampling period. Hence, the intra-IC RAMH treatment during CTA acquisition resulted in animals exhibiting a surge in ACh release during retrieval, as would be expected for a novel taste.

H_3_ receptor activation in the IC by RAMH during acquisition disrupted long-term CTA memory (Fig. 4D). Baseline water consumption did not differ among the groups (ANOVA *p*>.05; data not shown). A repeated measures ANOVA for the amount of saccharin solution consumed during acquisition and retrieval days revealed significant effects of group (F_1,8_ = 8.010 *p*<.05) and experiment day (F_1,8_ = 43.350, *p*<.01), and significant interaction between these two factors [F_1,8_ = 12.150, *p*<.01]. Fisher's *post hoc* tests indicated that saccharin consumption on the CTA acquisition day was similar between the groups (*p*>.05), indicating that the intra-IC RAMH injections during CTA acquisition had no effect on motivation or liquid consumption during conditioning. Nevertheless, *post hoc* analysis showed a significant difference in consumption between the groups during CTA retrieval (*p*<.01). Hence, intra-IC RAMH injections during CTA acquisition resulted in greater saccharin consumption during retrieval, indicating that the RAMH treatment impaired CTA memory formation.

### CTA-impairing pyrilamine injections into the NBM alter cortical ACh levels during CTA acquisition and retrieval

**Figure 5:**
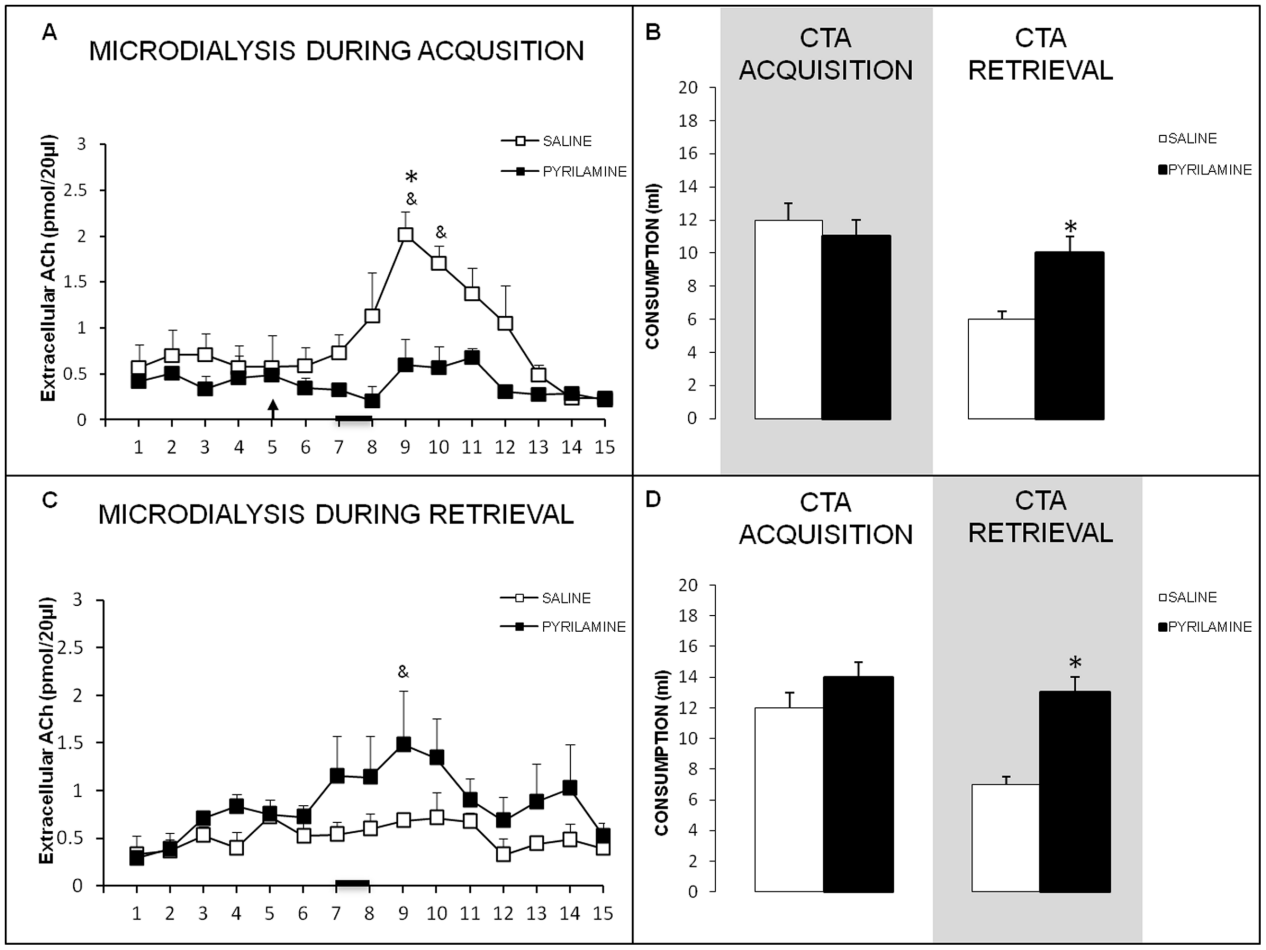
Pyrilamine effects in ACh release during CTA acquisition and retrieval. **A**) Extracellular ACh levels in the IC of free-moving rats during CTA acquisition. The arrow shows time of saline or pyrylamine infusion and black bar indicates saccharin consumption period (^&^ *p*<.05 vs. samples 5 and 6; *p<.05, between groups). **B**) Saccharin consumption during CTA acquisition (shading = microdialysis day) and memory retrieval (N = 5 for each group; *mean ± SEM*, *p<.05). **C**) Extracellular ACh in the IC of free-moving rats during CTA retrieval. The black bar indicates the saccharin consumption period (^&^ *p*<.05 vs. samples samples 1 and 2). **D**) Saccharin consumption during CTA acquisition (when infusions were administered) and memory retrieval (shading = microdialysis day). (N = 4 for each group; *mean ± SEM*, *p<.05).

Cortical ACh levels during CTA acquisition for the control and intra-NBM pyrilamine injected groups are presented in Figure 5A and 5C. A repeated measures ANOVA for ACh levels during the acquisition trial revealed significant effects of group (F_1,8_ = 6.159, *p*<.05) and sample (F_1,8_ = 6.343, *p*<.01), and a significant group×sample interaction (F_1,8_ = 3.176, *p*<.01). One way ANOVAs for each sample revealed that ACh levels differed significantly between the groups in sample 9 (F_1,8_ = 21.809, *p*<.01), which was collected during the last 15 min of the 45-min taste stimulus presentation.

The CTA behavioral data for the intra-NBM pyrilamine-infused animals and saline controls are presented in Fig. 5B. A simple ANOVA showed no significant differences between the groups in baseline water consumption (data not shown). A repeated measures ANOVA for saccharin solution consumption during acquisition and retrieval days, revealed experiment day significant effects (F_1,8_ = 23.120, *p*<.01), and significant interaction group×day (F_1,8_ = 6.480, *p*<.05), but not a significant effects of group (F_1,8_ = 3.368, *p*>.05) Fisher's *post hoc* tests indicated that saccharin consumption on CTA acquisition day did not differ between the groups (*p*>.05), demonstrating that pyrilamine injections into the NBM during CTA acquisition had no significant effect on motivation or liquid consumption during conditioning. However, during CTA retrieval, the pyrilamine injected group consumed more saccharin solution than did the saline control group (*p*<.05), indicating that the intra-NBM pyrilamine injections in the acquisition trial impaired subsequent CTA memory formation.

The ACh levels observed during memory retrieval in the control and pyrilamine-injected groups (injections during CTA acquisition and microdialysis only during retrieval) are shown in Figure 5C. A repeated measures ANOVA revealed a significant effect of sample on ACh levels during the retrieval trial (F_1,6_ = 2.793, *p*<.01), but no effect of group (F_1,6_ = 1.198 *p*>.05) and no significant group×sample interaction (F_1,6_ = 1.329, *p*>.05). Paired t-test for each sample revealed a significant increase of ACh in sample 9 compared with the basal ACh levels observed in samples 1 and 2 in pyrilamine group (*p*<.05).

H_1_ receptor blockade in the NBM by pyrilamine during acquisition disrupted CTA memory in the retrieval trial (Fig. 5D). Baseline water consumption did not differ between the groups (ANOVA, data not shown). Repeated measures ANOVA for saccharin solution consumption during acquisition and retrieval days revealed significant effects of group (F_1,6_ = 8.593, *p*<0.05) but not between experiment day (F_1,6_ = 5.143, *p*>.05), neither group×sample interaction (F_1,6_ = 2.286, *p*>.05). Fisher's *post hoc* tests showed that saccharin consumption on the CTA acquisition day did not differ between the groups (*p*>0.05), indicating that the intra-NBM pyrilamine injections did not affect motivation or liquid consumption during conditioning. Nevertheless, *post hoc* analysis showed that the pyrilamine-injected group consumed more saccharin solution during the CTA retrieval trial than did the saline controls (*p*<.05), indicating that pyrilamine injections into the NBM during acquisition impaired CTA memory formation.

## Conclusions

The main finding of the present research was the demonstration, in free-moving animals, of neurochemical modulation of ACh release into the IC being induced by local H_3_ receptor activation, or H_1_ receptor inhibition in the NBM, during CTA acquisition. This study demonstrates that the H_1_ and H_3_ receptor subtypes modulate ACh release in distinct ways, and that blocking ACh release into the IC by either manipulation during acquisition impairs subsequent CTA. Similar to observations in other cortical areas [30], [34], [35], [50]–[52], our immunofluoresence results showed that H_3_ receptors are expressed in approximately half of the GABAergic cells in the IC and that H_1_ receptors are expressed in most, if not all, cholinergic cells in the NBM. These results are in line with the knowledge that histaminergic axons, which originate solely from the tuberomamillary nucleus, innervate almost all brain regions [34], [51], [53], [54].

The present HPLC data are consistent with several previous studies demonstrating that novel stimuli induce cortical ACh release [5], [6], [28], [50], [55]–[58], and corroborate, in particular, prior work demonstrating that a novel taste (e.g. saccharin) increases ACh release within the IC of awake, freely moving rats, and that this release is dependent on the NBM [5]. Our findings also extend prior research demonstrating that ACh levels correlate with taste memory formation and that taste learning can be disrupted by NBM lesions [59] or cortical cholinergic antagonism [60], and can be enhanced by cholinergic agonism in the IC [15]. This convergence of evidence indicates that cholinergic activity in the insular gustatory neocortex, particularly cholinergic activation coming from the NBM, plays a critical role in the mnemonic representation of taste [61]–[64].

The present findings support the hypothesis that ACh modulates the general efficacy of sensory cortical processing during information association [21]. However, important questions arise from this hypothesis. For example, if cholinergic neuromodulation participates in memory formation, either by encoding novelty at the cellular level, or by instructing the neural circuits to store the novel taste representation, it is not clear how cholinergic feedback activity is mediated in the NBM during novelty or familiarity recognition. Additionally, it is not known how the selective pathways that originate in the NBM and are involved in taste memory formation are controlled. In this regard, an increasing number of studies are providing new evidence suggesting that the histaminergic system could have an important function during the interactions mediated by NBM cholinergic activity. It has been demonstrated that the histaminergic system is largely responsible for cortical activation and cognitive activities during wakefulness [65]; it also constitutes an important wake-promoting system [66] and participates in the complex regulation of sleep stages, feeding, and cognition [35], [45], [67], [68]. Given that the NBM receives substantial histaminergic afferents from the tuberomamillary nucleus [33], [34], [69]–[71], the functional specificity of histamine release during cognitive processes may depend on the brain regions involved and the histaminergic receptor subtypes being bound, as well as on the nature of the cognitive task [72], [73].

A second important set of findings from this study was that injections of the H_3_ agonist RAMH into the IC or of the H_1_ antagonist pyrilamine into the NBM impaired taste aversive memory formation while decreasing ACh levels in the IC, which are normally elevated during novel taste consumption. These results indicate that increased cortical ACh release during novel taste consumption requires the integrity of (at least) two subtypes of histaminergic receptors localized in the NBM and IC. Furthermore, our results show that opposing actions by cortical H_3_ receptors and basal H_1_ receptors are crucial for novelty processing during CTA acquisition. These findings agree with previous reports in which blockade of NBM histamine receptors impaired memory formation of different tasks that require an intact cholinergic system [52], [74], [75]. Moreover, H_3_ receptor activation facilitates object recognition memory and aversive context learning [30], [76], [77], suggesting that monoaminergic-cholinergic system interactions [78] are modulated by H_3_ receptors that regulate ACh release in the entorhinal cortex [51], [79], NBM, and medial septum [50], [80]. Additionally, NBM cholinergic neurons have been shown to be activated mainly through H_1_ histamine receptors, and histamine injections into the basal forebrain have been shown to increase ACh release in the cortex [6], [31], [34], [37], [43], [74].

Recent evidence showing opposite effects of different histamine receptor subtypes highlights the complexity of the histaminergic system [32], [40], [63]. Bacciottini et al. (2001) have posited that the histaminergic system may be comprised of two components: one inhibitory, related to local nerve terminal actions, and the other excitatory, interacting with cholinergic cells in the cholinergic basal forebrain. H_3_ receptors, located post-synaptically in the cortex, facilitate GABA release, which appears to inhibit increases in ACh release in the cortex [52]. Inhibition of ACh release by H_3_ agonism in the cortex can be reversed by local GABA_A_ receptor antagonism [81]. Conversely, histamine in the NBM acts in the opposite fashion, facilitating the release of cortical ACh through H_1_ receptors [32], [74]. For example, intra-NBM administration of histamine through a microdialysis probe increases ACh release in the parietal cortex, probably through H_1_ receptors [6], [50]. In light of the aforementioned evidence of dual, opposing effects of histamine—excitatory on NBM cell bodies and inhibitory on cholinergic terminals [30], [81]—our demonstration here that GABAergic cells in the IC express H_3_ receptors suggests that there may be an important GABA/ACh interaction during taste memory formation that is regulated by histaminergic activity. Further studies are needed to test this hypothesis.

The present evidence indicates that H_1_ receptors in the NBM and H_3_ receptors in the IC must be activated and inhibited, respectively, during novel taste learning, since the pharmacological manipulation of these receptors can alter release of ACh into the IC, and this release is a requirement for novel processing during taste learning. Our hypothesis also includes the possibility that GABA activity could be modulated by histamine receptors in the IC during acquisition as well as during retrieval of taste aversive memory [11], [82].

Finally, accumulating evidence shows that histamine plays a major role in the maintenance of arousal and contributes to the modulation of appetite, energy homeostasis, motor behavior, and cognition [40], [68], [83], [84]. All of these diverse physiological roles are involved in the complex task of feeding, a major guiding influence of which is taste memory. The evidence presented in this article indicates that pathways modulated by NBM cholinergic activity are coordinated by different subpopulations of histamine receptors located on the cell body or terminals, providing further support for the view that “the histaminergic system is organized into distinct functional pathways modulated by selective mechanisms” [32]. In particular, during taste memory formation and retrieval, ACh release into the cortex may be finely tuned by histaminergic activity to coordinate novel versus familiar stimulus processing in taste memory recognition.
